# Assessing the Neuronal Serotonergic Target-based Antidepressant Stratagem: Impact of *In Vivo* Interaction Studies and Knockout Models

**DOI:** 10.2174/157015908785777256

**Published:** 2008-09

**Authors:** R Rajkumar, R Mahesh

**Affiliations:** Pharmacy Group, FD-III, Vidya Vihar, Birla Institute of Technology & Science, Pilani, Rajasthan-333031, India

**Keywords:** Serotonin, depression, trophic factors, interaction studies, knockout models, preclinical screening, target.

## Abstract

Depression remains a challenge in the field of affective neuroscience, despite a steady research progress. Six out of nine basic antidepressant mechanisms rely on serotonin neurotransmitter system. Preclinical studies have demonstrated the significance of serotonin receptors (5-HT_1-3,6,7_), its signal transduction pathways and classical down stream targets (including neurotrophins, neurokinins, other peptides and their receptors) in antidepressant drug action. Serotonergic control of depression embraces the recent molecular requirements such as influence on proliferation, neurogenesis, plasticity, synaptic (re)modeling and transmission in the central nervous system. The present progress report analyses the credibility of each protein as therapeutically relevant target of depression. *In vivo* interaction studies and knockout models which identified these targets are foreseen to unearth new ligands and help them transform to drug candidates. The importance of the antidepressant assay selection at the preclinical level using salient animal models/assay systems is discussed. Such test batteries would definitely provide antidepressants with faster onset, efficacy in resistant (and co-morbid) types and with least adverse effects. Apart from the selective ligands, only those molecules which bring an overall harmony, by virtue of their affinities to various receptor subtypes, could qualify as effective antidepressants. Synchronised modulation of various serotonergic sub-pathways is the basis for a unique and balanced antidepressant profile, as that of fluoxetine (most exploited antidepressant) and such a profile may be considered as a template for the upcoming antidepressants. In conclusion, 5-HT based multi-targeted antidepressant drug discovery supported by *in vivo* interaction studies and knockout models is advocated as a strategy to provide classic molecules for clinical trials.

## DEPRESSION: DIAGNOSIS AND PHARMACOLOGICAL PERSPECTIVE

Antidepressant drug discovery has been a complex task owing to incomplete understanding of neurobiological basis of depression. Continuous identification of new biomarkers and proteins has desperately given shape to the neural picture of this group of disorder. However no single molecular target can be finalized as an ultimate therapeutic strategy. Depression is classified under mood disorder and has many subtypes. Identification of symptom clusters (vegetative, cognitive, impulse control behavioural and somatic) have visualised depression as a syndrome [290]. Though diagnostic criteria [9] has been continuously subjected to refinement, incidence of different overlapping symptoms and subtypes (their neural correlates), co-morbidity with other psychiatric [80, 248] and /or terminal illnesses [84,147,197] obscured the treatment lines. As a result, management of depression has been merely symptomatic, demanding poly-pharmacy and/or chronic drug therapy. Intensive research at both preclinical and clinical levels has still left us with treatment regimen offering patient recognizable improvement only after several weeks of treatment [3, 11, 30, 224]. Such a scenario encourages new drug discovery aimed at producing new molecules with faster onset and reduced tolerance. Identification of various neuronal targets and screening of molecules from different chemical classes lead drug discovery programs to launch a wide variety of drugs (with different pharmacokinetic profiles). Unravelling the complex neural circuitry of depression should essentially run in parallel, to fine tune the existent antidepressant drug discovery programs. Superficially, harmony among neurotransmitter systems is expected from an ideal antidepressant. The presently available drugs mainly exploit the amine hypothesis of depression, principal locus of action being the serotonergic and/or norepinephrine neuron and with a secondary importance to the dopamine neuron. 

**Fig. (1):**
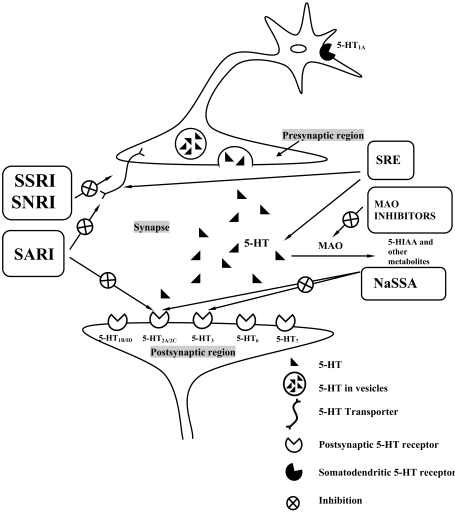
Schematic representation of different antidepressant mechanisms at the serotonergic synapse. The diagram depicts only those possible sites of action which have been associated with the molecular basis of depression till date. The SSRIs, SNRIs and SARIs inhibit 5-HT reuptake by acting on the transporter. SARIs also block the postsynaptic 5-HT_2_ receptors. MAO inhibitors prevent 5-HT breakdown thereby increasing the synaptic concentrations. NaSSAs (α_2_ antagonists) block postsynaptic 5-HT_2_ and 5-HT_3_ receptors. SREs, enhance the presynaptic 5-HT uptake and also prevent the susceptibility of 5-HT to MAO.

The antidepressant mechanisms of drug action have been channelled down to nine ‘pharmacological routes’ [332]. Six of the nine basic antidepressant mechanisms involve the serotonergic system and are explained as follows (Fig. **1**). The selective serotonin reuptake inhibitors (SSRIs), serotonin norepinephrine reuptake inhibitors (SNRIs) and serotonin antagonist and reuptake inhibitors (SARI) share a common mechanism of inhibiting the serotonin trasporter. SARIs, inaddition have 5-HT_2_ receptor antagonistic property which reduces the side effects associated with the treatment. Noradrenergic and specific serotonergic antidepressants (NaSSA), apart from α_2_ receptor blockade, also antagonize 5-HT_2_ and 5-HT_3_ receptors. Monoamine oxidase (MAO) inhibitors prevent degradation of 5-HT, compensating for the decrease in synaptic 5-HT observed in depression. Serotonin reuptake enhancers (SREs) which possess a reverse mechanism to that of reuptake inhibitors, have lead to new way of antidepressant action. As an additional mechanism, they decrease the susceptibility of 5-HT to MAO. The effects on 5-HT receptor subtypes not only reduce the side affects associated with antidepressant treatment, but also inspired us to check whether these receptors can influence the disorder itself. Present developments in the fields of molecular pharmacology and biotechnology revealed the role of gene activation/suppression, protein expression, neuronal plasticity, and neurogenesis and various other inter-linked complex phenomenon associated with behavioural and neurochemical states of depression. This review updates the identified and probable neuronal targets of depression mainly pertaining to the 5-HT neurotransmitter system and attempts to highlight the significance of knockout models and interaction studies in both identifying new targets and screening specific ligands (acting directly/indirectly on the serotonergic system) for antidepressant prospects.

## SEROTONERGIC SYSTEM AS DIRECT ANTIDEPRESSANT DRUG TARGET

Several reviews have recognized the pivotal status of serotonergic system in depression and antidepressant drug action [25, 270, 310]. 5-HT as a central dogma of depression, is involved in synaptic plasticity, focal and/global neurogenesis [224] and many other intricate neurogenetic mechanisms [146,164,167]. The preclinical studies which attempted to clarify the role of serotonergic receptors, the secondary functional proteins and various other factors involved in control of depression are henceforth discussed.

## 5-HT_1A_ RECEPTOR

This somatodedritic autoreceptor in the raphae nuclei [240,258] influences neuronal firing, 5-HT synthesis and release [29, 126,134,156]. The presence of postsynaptic 5-HT_1A_ receptors in the limbic structures viz. hippocampus, amygdala and frontal cortex (of rats and primates) [14] is suggestive of their role in depression [45]. 8-hydroxy-2-(din-propylamino)tetralin (8-OH-DPAT), a selective 5-HT_1A_ agonist induces hypothermia in mice [99] and rats [100]. Many interaction studies employed 8-OH-DPAT to examine the 5-HT_1A_ mediated antidepressant effects of test substances [47, 176, 72, 36]. Adenylate cyclase inhibition assay in rat hippocampal membranes vaguely predicted that antidepressant-like effects of 8-OH-DPAT (and buspirone) are mediated by postsynaptic action involving the serotonergic second messenger transduction in hippocampus [227]. 8-OH-DPAT is also known to influence dopamine release [4,135]. Involvement of 5-HT_1A_ receptors *per se* has been evidenced in various interaction studies in different models of depression. Agonists of 5-HT_1A_ receptor were shown to exhibit antidepressant-like effects in rodent forced swim test (FST) [234, 281, 323], discriminative taste aversion test [175], learned helplessness behaviour test in rats [96], and in bulbectomised rats [204]. Flesinoxan, a specific 5-HT_1A_ agonist has exhibited antidepressant-like effects in three models of depression viz. FST, 8-OH-DPAT induced hypothermia and olfactory bulbectomy [63]. Altered function of 5-HT_1A_ receptors was reported in olfactory bulbectomised rats, a model of chronic depression [106]. Microdialysis study in rats indicated that antidepressant-like effects of serotonergic drugs were potentiated by 5-HT_1A_ autoreceptor blockade [260]. 5-HT_1A_ function is also involved in effectiveness of electroconvulsive shock treatment as observed from and hypothermic responses in rats [329] and electrophysiological analysis of rat hippocampal slices [139]. Normalization of 5-HT synthesis was associated with antidepressant-like effect of chronic buspirone (5-HT_1A_ partial agonist) treatment [320]. This has emphasized the role of 5-HT_1A_ receptor in the pathophysiology of depression, adhering to the monoamine theory. Faster onset was evident when SSRIs and 5-HT_1A_ antagonists were combined [11,61,62] in giving the first set of clues to 5-HT_1A_ receptor involved in SSRI drug action. Furthermore, it has been found that antidepressant-like effects of SSRIs were mediated by the activation of 5-HT_1A_ receptors [125,295] which alter the responsiveness of receptor-mediated G-protein-coupled inwardly rectifying potassium (GIRK) currents [59]. 5-HT_1A_ knockout mice exhibited decreased baseline immobility in forced swim and tail suspension tests (TST) [120, 203] indicating the pivotal role of this receptor, in depression. Only under chronic stress conditions, the 5-HT_1A_ receptor mRNA is modulated by chronic antidepressant treatment, indicating the occurrence of multiple pathways associated with the interaction of stress and drug treatment [1]. Thus, modulating 5-HT_1A_ receptor is definitely beneficial in depression, providing in addition, a faster onset of action.

## 5-HT_1B_ RECEPTOR

It is a presynaptic heteroreceptor [269,268] expressed in nucleus accumbens, caudate putamen, dorsal raphe nucleus and some cortical areas [42, 269]. In guinea pigs the 5-HT_1B_ mRNA was shown to be widely distributed throughout the brain, especially in the striatum, nucleus accumbens, olfactory tubercle, cortex, hypothalamus, hippocampal formation, amygdala, thalamus, dorsal raphe and cerebellum [33]. RU 24969, a 5HT_1B_ agonist was shown to reduce the hippocampal 5-HT efflux [194] and blockade of presynaptic 5-HT_1B_ receptors enhanced the SSRI’s induced 5-HT release in rats [68]. Moreover fluoxetine reversibly reduced 5-HT_1B_ mRNA in rat dorsal raphae nucleus [10, 226] suggesting the possible influence of 5-HT_1B_ receptor on depressive states. The antidepressant-like effects of venlafaxine, an SNRI, are likely to involve 5-HT_1B_ receptors [256]. Following the aforesaid observations, antagonists of 5-HT_1B_ receptor were screened for antidepressant potential. GR 127935, a non-selective 5-HT_1B_ antagonist, reversed the antidepressant-like effects of paroxetine [92] and was inactive in mice FST [296]. On the contrary, the molecule advanced the onset of antidepressant-like effects of fluoxetine in rat schedule induced polydipsia test [128]. A study based on thermoregulatory responses indicates the existent functional interaction between 5-HT_1B _and 5-HT_1A_ receptors [91]. Studies with knockout mice have shown that presynaptic receptors limit the ventral hippocampal 5-HT release, following the chronic paroxetine treatment in mice [90, 186]. 5-HT_1B_ deletion failed to affect the baseline immobility but increased sensitivity to fluoxetine in TST [203] and paroxetine was inactive in knockout mice [90]. Among the 5-HT_1B_ knockout mice, females demonstrated higher baseline levels of hippocampal 5-HT depletion which was reasoned as sex-linked disinhibition [145]. Presently, 5-HT_1B_ receptor (originally found only in rodents) was reported to be expressed in human brain [27, 312, 313] and increased 5-HT_1B_ mRNA was associated with bipolar disorder [172]. In a nutshell, preclinical testing of 5-HT_1B_ ligands has left us with inconclusive results whereas knockout studies indicated that antagonism of 5-HT_1B_ receptor may help antidepressant drug action. Thus, co-localization with 5-HT_1A_ subtype in key regions involved in depression was the only feature linking its etiological role.

## 5-HT_1D_ RECEPTOR

This sparsely distributed receptor is co-localized with that of 5-HT_1B_ receptor [41,42], in olfactory tubercle, entorhinal cortex, dorsal raphe, cerebellum, mesencephalic trigeminal nucleus and in the trigeminal ganglion [33]. LY393558, a nonselective 5-HT_1B/1D_ antagonist increases the extracellular 5-HT levels in guinea pig hypothalamus and rat brain [211]. None of the selective 5-HT_1D_ ligands have found its use in depression and hence 5-HT_1D_ receptor is highly improbable as a neuronal target. However, while designing a prospective antidepressant, 5-HT_1D_ antagonism would be desirable to facilitate faster onset [270].

## 5-HT_2A_ RECEPTOR

**Fig. (2):**
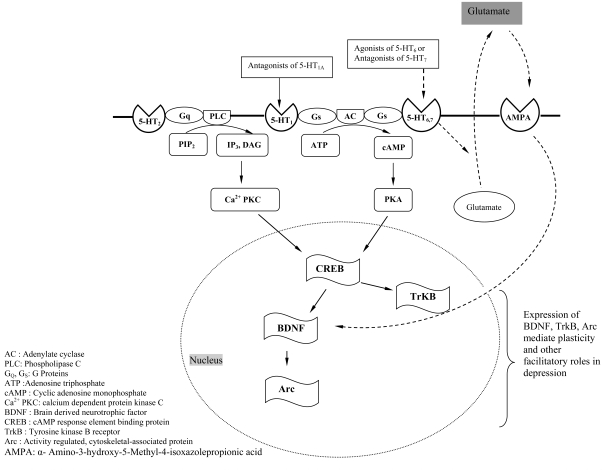
A simplified schematic representation of cellular events involving the neuronal metabotropic 5-HT receptors associated with depression. 5-HT_2,6,7_ subtypes are post-synaptic receptors, whereas 5-HT_1A_ is a somatodendritic autoreceptor. To simplify the cellular events, all the receptors (somatodendritic and post-synaptic) have been depicted in a single diagram. The 5-HT_1, 6, 7_ and 5-HT_2_ receptors through cAMP-PK and IP3–Ca^2+^ dependent protein kinase secondary messenger systems influence CREB.  The agonists of 5-HT_6_, or the antagonists of 5-HT_7_ facilitate glutamate release. Increase in extracellular concentration of glutamate activates BDNF through AMPA receptors. BDNF and TrkB are target genes for CREB (transcription factor). BDNF in turn activates Arc. Altered expression of BDNF, CREB, TrkB and Arc are of potential benefit in depression. (Based on references [77] and [230]).

This subtype is found in cell bodies and processes of neurons in the hippocampus, amygdala, striatum, olfactory structures. Their presence in glial structures is expected to have functional implications [328]. These receptors are co-localized (80%) with 5-HT_1A_ receptors and their mRNA is expressed in the rat and mouse prefrontal cortex [8]. Antidepressants of almost all pharmacological classes predominantly down-regulate 5-HT_2A_ receptors [98, 243] through various mechanisms [302], inspiring us to consider this receptor as a potential target. Assessment of various serotonergic agents in differential-reinforcement-of-low-rate 72-sec behaviour in rats, has lead to a hypothesis that both 5-HT_1A_ and 5-HT_2_ receptors (agonism of 5-HT_1A_ receptor and antagonism of 5-HT_2_ receptor) are clinically significant to arbitrate antidepressant effects of drugs [191]. Supporting this hypothesis, it has been demonstrated that chronic corticosetrone treatment induces depression-like behavioural manifestations in rodents with decreased 5-HT_1A_ and increased 5HT_2A_ receptor binding [83]. Chronic antidepressant treatment alters 5-HT_2A_ (and 5-HT_2C_) mediated hyperthermia in Fawn-Hooded rats (genetic model of depression) [13]. Antisense oligonucleotide induced 5-HT_2A_ receptor down-regulation is sufficient to achieve antidepressant-like effects in mice and enhances psychomotor activity. BDNF, a trophic factor (discussed henceforth) has been linked with 5-HT_2A_ receptors (Fig. **2**). The antidepressant-like effects of desipramine in bulbectomised rats are correlated with decrease in 5-HT_2A_ receptors of frontal cortex [218]. In mouse FST, antidepressant-like effects of imipramine and desipramine can be partly attributed to antagonism of 5-HT_2A_ receptors [255]. The norepinephrine activity due to SSRI and 5-HT_2A_ antagonism is expected to provide benefit in affective disorders [292]. Drugs with selectivity to the 5-HT_2A_ receptor could be used to manage certain symptoms of depression [280], if not all. Specific 5-HT_2A_ receptor antagonists down-regulate BDNF mRNA expression in rat hippocampus and neocortex [307]. Further, the activation of 5-HT_2A_ receptor has a beneficial effect on 5-HT induced de novo BDNF mRNA synthesis and this is mediated by a calcium and protein kinase-dependent pathway [206]. The direct role of 5-HT_2A_ receptor in depression and facilitatory effects on SSRI action are well reported in the literature [45, 251]. The criterion of 5-HT_2A_ antagonism for antidepressant action has triggered the consideration of experiments screening 5-HT_2A_ antagonistic potentials in the test batteries for new compounds. Many selective 5-HT_2A_ antagonists, namely, HT-90B [138, 305], YM-992 [293], EMD 281014 [238] and M100907 [192], have shown antidepressant-like outcomes in animal models. However lack of adequate data from knockout studies have made the involvement of 5-HT_2A_ receptors in depression questionable.

## 5-HT_2C_ RECEPTOR (Previously 5-HT_1C_ Receptor)

The pharmacological equivalence of 5-HT_1C_ with 5-HT_2C_ receptor has lead to a change in nomenclature to the latter [249]. Studies on 5-HT_2C _receptor distribution (the intial studies on distribution reported as 5-HT_1C_) in rat CNS, revealed that this receptor is abundant in the anterior olfactory nucleus, medial and intercalated amygdaloid nuclei, hippocampus layers CA1 to CA3, latero-dorsal and lateral geniculate thalamic nuclei, caudate-putamen and several areas of the cortex [54, 127, 207,  212, 249]. The aforementioned 5-HT_2C_ receptor locations were consistent in rats and mice; most of these the areas being involved in depression. The first set of evidences linking this receptor and depression originated when antidepressants of different chemical classes mianserin [173, 239] and imipramine [35,142] were linked to depressive states in rats. The functional interaction with 5-HT_1A_ receptors [22] may be critical for the antidepressant-like effects [21, 143]. Clorgyline treatment significantly reduces [^3^H] mesulergine binding in rat hypothalamus and striatum which is suggestive of the involvement of 5-HT_2C_ receptors in the clinical efficacy [40]. High affinity selective agonists, Ro 60-0175 and Ro 60-0332 have been proved efficacious in various rodent (rat) models of depression viz. stress induced anhedonia [216],olfactory bulbectomy induced passive avoidance, operant tasks, and electroencephalographic studies [141, 193], and in FST [60]. In Fawn-Hooded rats short-term lithium treatment enhances 5-HT concentrations at postsynaptic 5-HT_2C_ receptor sites [12]. Amidst the various mechanisms behind the antidepressant effect fluoxetine, a competitive and reversible antagonism at 5-HT_2C_ receptor was reported [48, 229] . Selective 5-HT_2C_ receptor agonists WAY 161503, RO 60-0175 and RO 60-0332 decreased immobility and increased swimming in modified rat FST [60]. In addition, effects of fluoxetine were blocked by SB 206533 (a selective 5-HT_2C_ antagonist) and synergized by selective agonist, RO-60-1075 [55] indicating that 5-HT_2C_ receptor has significant implication on therapeutic effects of antidepressants. Hence, it is observed that agonistic effects at 5-HT_2C_ may be beneficial in depression.

## 5-HT_3_ RECEPTOR

The electrophysiologically characterized 5-HT_3_ receptors [245] are found in median raphae, hypothalamus, hippocampus, amygdala [148, 149, 161, 316], which and neural correlates of depression. Several preclinical (behavioural, neurochemical and genetic) investigations have provided evidences linking 5-HT_3_ receptors and depression. Serotonin type-3 receptor antagonists (5-HT_3_ RAs) reverses escape the deficits in rat learned helplessness test [195], a sensitive antidepressant screening method. Antidepressants such as fluoxetine [82], imipramine, phenelzine and iproniazid [81] inhibit the 5-HT current mediated by 5-HT_3_ receptor in rat nodose ganglia. Antidepressant drugs that increase 5-HT release through a 5-HT_3_ mechanism, modulates noradrenaline levels [214]. Selective 5-HT_3_ receptor antagonist ondansetron alters local cerebral glucose utilization (LCGU) in the rat median raphe [210], potentiated anti-immobility effects of SSRI’s [255] indicating the role played by 5-HT_3_ receptors in depression. In mice FST, it has been observed that antidepressant-like effect of reuptake inhibitors is associated with potassium ion (K^+^) - channel linked 5-HT_3_ receptors [38, 105, 257]. Though studies contradicting the aforementioned concept have been reported [34, 35, 132, 176], knockout mice have exhibited sex dependent differences of depressive states in FST [26]. Other studies with 5-HT_3_ receptor antagonists such as ICS 205-930 [222], MDL 72222 [153] and tropisetron [39] indicate the significance of 5-HT_3_ receptors in depression. Recently, research carried out in our laboratory revealed antidepressant-like effects of a substituted naphthyridine carbonitrile [185] which possessed a pA_2 _value comparable to ondansetron in tissue based assay [184]. The antidepressant-like effects of chronic ondansetron were demonstrated in a battery of rodent assays and involvement of post-synaptic 5-HT_3_ receptor was speculated [252]. The 5-HT_3_ antagonism which was known to alleviate the side effects of antidepressant treatment is now being considered to compliment drug action.

## 5-HT_6_ RECEPTOR

Studies using various biochemical techniques such as histochemistry, polymerase chain reaction and northern blot have elucidated the undoubted presence of these receptors in a list of depression related neural structures (limbic system), nucleus accumbens, olfactory tubercle, hippocampus and hypothalamus [94, 215, 263, 319], with an apparent co-localisation with 5-HT_2A/2C_ receptors in rat striatum [318]. Apart from cloning, functional and binding characterization [31, 284], the receptor binding affinities with various antidepressants have been studied [215]. Hence this adenlyl cyclase-cAMP coupled receptor [263, 272] is now viewed as a prospective target for antidepressant drug action. Interaction studies with various selective 5-HT_6_ ligands in different assay systems have been carried out to study the effects on depression. The expressions of phospho-Ser845-GluR1 and c-fos mRNA (in striatum and cerebral cortex) are enhanced by administration of fluoxetine or 2-ethyl-5-methoxy-N,N-dimethyltryptamine (EMDT), a 5-HT_6_ receptor agonist. SB 271046, a selective 5-HT_6_ antagonist though inactive when administered alone, is found to reverse the stimulatory effects of fluoxetine and EMDT. The compound also reverses the antidepressant-like effects of fluoxetine and EMDT in mice TST [291]. In contrast to the aforesaid activity, SB-399885 (selective 5-HT_6_ receptor antagonist) has demonstrated anti-immobility effects in forced swim (in both rats and mice) and TST (in mice) without influencing motor coordination and behaviour. However the antidepressant-like effects are said to be mediated by non serotonergic mechanism [321]. Intra-hippocampal administration of SB 258585 (5-HT_6_ receptor antagonist), has shown anti-immobility effects in rat FST [289]. The selective 5-HT_6_ receptor agonist LY-586713, up-regulates (with a bell-shaped dose response) hippocampal BDNF mRNA expression and increases the cortical and hippocampal levels of activity regulated, cytoskeletal-associated protein (*Arc*). Pre-treatment with the selective 5-HT_6_ receptor antagonist SB-271046 completely blocks this up-regulation in BDNF and attenuates *Arc* levels in the respective regions (observed at active dose level of LY-586713). BDNF up-regulation has been through sequential activation of cAMP and CREB (transcription factor for BDNF gene) or due to increase in extracellular glutamate induced by 5-HT_6_ receptor activation (Fig. **2**). Similarly, the increase in *Arc* mRNA expression could be linked to cAMP, glutamate and BDNF [70]. In summary, these reports confirm the beneficial effects of 5-HT_6_ receptor stimulation in managing depression.

## 5-HT_7_ RECEPTOR 

This receptor was discovered by targeted cloning strategies and the localization and pharmacology of 5-HT_7_ receptors have been the initial clues [276]. This sparked antidepressant screening studies with specific ligands to associate them with serotonergic depression. High receptor density is identified in the medial thalamic nuclei and related limbic and cortical regions of guinea pig brain [300]. The four isoforms of this receptor has been found to be pharmacologically equivalent and are distributed in amygdala, cortex, hippocampus, thalamus, septum, hypothalamus and suprachiasmatic nucleus of rat brain [109]. Distribution of this receptor has been extensively studied in different species [109, 118, 300, 276, 297, 298] and it is present at intermediate levels in analogous human brain areas [314]. Chronic fluoxetine treatment down-regulated the hypothalamic 5-HT_7_ receptor binding sites in rats [285] and acute restraint stress up-regulated 5-HT_7_ mRNA expression in rat hippocampus [330] indicating the participation of 5-HT_7_ receptor in depression. Many antidepressants have attenuated c-FOS expression which is consistent with activation of 5-HT_7_ receptor in suprachiasmatic nucleus [219]. Prototypic antidepressants have been antagonists at enteric 5-HT_7_ receptors acting through allosteric or competitive mechanisms [174]. SB-656104-A (5-HT_7_ antagonist), has been found to reduce the time spent in REM sleep in rats [299]. Non-selective 5-HT_7_ agonist 5- carboxytryptamine (5-CT) induced hypothermia in guinea-pigs [112] and mice [108] but fails in 5-HT_7_ receptor knockout mice [108, 116].

Research based on knockout and interaction studies, which have been conducted in the recent past, have provided us with substantial evidence pertaining to the role of 5-HT_7_ receptors. 5-HT_7_ knockout mice exhibit antidepressant-like effects in FST. Attenuation of circadian rhythm phase shifts to 8-OH-DPAT, observed in extra-cellular recordings from hypothalamic slices (5-HT_7_ knockout) and failure of SB-258719 (a selective 5-HT_7_ receptor antagonist) as an antidepressant (in FST using wild mice) in light phase signifies the circadian cycle dependent activity [107]. Furthermore, 5-HT_7_ knockout mice have been screened in behavioural antidepressant assays namely, FST, TST and REM sleep pattern analysis [117], which have eventually strengthened the hypothesis that inhibition of 5-HT_7_ receptors could be beneficial in depression [118]. In a series of studies conducted in mice, SB-269970 decreases the duration of immobility in FST and TST with a characteristic ‘U’ shaped dose response curve [322], augments antidepressant-like effects of citalopram in TST indicating a pharmacodynamic interaction [32] and antidepressant-like effects. As far as the REM sleep studies are concerned, the antidepressant-like effects of 5-HT_7_ receptor antagonists are accepted with some degree of uncertainty since the sleep pattern observed in rats and mice is in contrary to what has been observed in clinical depression. 5-HT_7_ receptor is involved in shaping of neuronal cytoarchitecture, especially the hippocampus and the antidepressant-like behavioural effects due to 5-HT_7_ antagonism can be possibly associated with altered morphology and neurogenesis [223]. Blockade of endogenous corticosterone biosynthesis triggers up-regulation of hippocampal 5-HT_6_ and 5-HT_7_ mRNA expression and such an effect has been partially reversed by corticosterone replacement. This up-regulation partly explains the earlier reported therapeutic actions of adrenal steroid synthesis inhibitors in resistant depression [331]. Inhibition of the central 5-HT_7_ receptor appears to be useful in depression, supporting them as potential targets.

## OTHER PROBABLE TARGETS LINKED TO 5-HT SYSTEM

### Trophic Factors

Research has clarified the mysteries behind the BDNF and cAMP-CREB neurotrophic systems and has led us to a new way in understanding the depressive disorder simultaneously providing new targets for drug action [77]. While arriving at the molecular and cellular hypothesis of depression behind the apparent neurotransmitter theory, several composite intracellular mechanisms [188] which amplify the neurotrophic factors [75,294] eventually regulate the life, structure and functioning of neurons [76]. The following section is expected to clarify how these factors are linked to serotonergic system *per se* and to review the existence of other factors linked to this system.

### Brain Derived Neurotrophic Factor and Tyrosine Kinase B Receptor

Tyrosine Kinase B (TrkB) receptors are present in serotonergic neurons of the raphe nuclei and their ascending projections into the dorsal hippocampus [181], neostriatum and nucleus accumbens [86], in rat brain. Biarylpropylsulfonamide AMPA receptor potentiator (LY404187) and its active isomer (LY451646) showed a dose and time-dependent modulation of BDNF and TrkB mRNA expression [180]. The endogenous ligand of TrkB receptor is the brain derived neurotrophic factor (BDNF). Central administration (in rats) of BDNF in Midbrain [282] and dentate gyrus of Hippocampus reversed escape deficits in learned helplessness test and decreased duration of immobility in FST without influencing locomotor status [278]. Down-regulation of the (Trk-B)-BDNF pathway is observed in major depressive disorder [303]. BDNF and its receptor TrkB are modulated by drugs of other pharmacological classes. To mention a few, antidepressant-like effects of delta-opiod receptor agonist (+)BW373U86 [301], Deltamethrin, a pyrethroid alkaloid [304] were found to be mediated by BDNF., which demonstrate the antidepressant-like effects. In the contrary, intraventral tegmental area infusion of BDNF induced depression like states [79]. Studies targeting the TrkB receptor revealed that despite TrkB deficient mice are not suitable as model of depression [339], its over-expression in mice induces resistance to behavioural despair [152].

Proclaimed as the *‘dynamic duo’* and together controlling the homeostasis against depression [201], the liaison between them BDNF and 5-HT has been strengthened by pharmacological [5, 77, 200, 282] and stem cell based research [20, 241]. BDNF helps in the neurogenesis and neuroprotection of 5-HT neurons [187] and alters 5-HT receptor expression in different regions of mice brain [177]. 5-HT acting through the 5-HT_1A_ autoreceptor, up-regulates BDNF which eventually acts on the TrkB receptor [88]. 5-HT_2A_ blockade can produce restraint stress induced BDNF suppression [307, 308], correspondingly its activation increases the de novo BDNF mRNA synthesis [206]. A study using different 5-HT enhancing ligands indicated that an increase in 5-HT, induced a ligand dependent and region specific modulation of BDNF mRNA levels in rat brain [336]. Amitryptaline and venlafaxine treatment increased BDNF levels particularly in the hippocampal neurons [327] and similar effects were observed in other areas when treated with desipramine and tranylcypromine [115]. Such activation is due to a cascade of events involving indirect stimulation of neurotransmitter receptors probably via increases in endogenous 5-HT levels in synapses of specific brain regions [37]. Fluoxetine also protected dexamethazone induced neuronal damage by augmenting BDNF levels [115], especially in dopaminergic neurons [213]. A biphasic pattern is observed in the BDNF gene expression that justifies the slow onset of action commonly observed with SSRIs [58]. Fluoxetine exhibits duration of treatment dependent influence on BDNF expression [69]. Paroxetine, a well tolerated SSRI enhances synaptic plasticity in the hippocampus by boosting BDNF mRNA expression [196]. The recent approach in the understanding of antidepressant drug action involves the concepts of adaptation or plasticity of neural systems and BDNF is found to play a profound role in it [65]. Chronic restraint stress negatively influences the BDNF expression which was blocked by chronic administration of quetiapine or venlafaxine, the effects being potentiated when combined [326]. Chronic corticosterone in addition to the influence on the 5-HT system, has been shown to impair hippocampal BDNF function, which is comparable with the hippocampal atrophy reported in major depression [140]. Social aversion (one of the depression related behaviour) in mice is mainly influenced by BDNF-regulated molecular pathways in the NAc and is counteracted by antidepressant drugs especially fluoxetine [24]. Though hippocampal BDNF expression was not influenced with wheel running behaviour (a model of learned helplessness) in rats [101], increased BDNF and decreased 5-HT turnover was observed in frontal cortex and hypothalamus in olfactory bulbectomised mice, a sensitive rodent model of hyposerotonergic depression [121]. An *in vitro* study probing the drug-modulation effects in neural stem cells (adult rat hippocampus) indicated that imipramine promotes serotonergic differentiation via the modulation of the BDNF/MAPK/ERK pathway/Bcl-2 cascades [241]. 5-HT_1A_ receptor function was attenuated in the dorsal hippocampus of BDNF knockout mice [122]. As observed from the recent reports the 5-HT-BDNF interaction supports the monoamine theory of depression and is expected to provide new insights in relation to faster onset refining antidepressant therapy.

### Cyclic Adenosine Monophoshate Response Element Binding Protein 

Cyclic AMP response element binding protein (CREB) is a nuclear protein belonging to a family of leucine zipper transcription factors. As the name suggests, it involves the cAMP cascade and cAMP dependent protein kinase for activation and phosphorylation respectively [95, 209]. BDNF itself is a target gene regulated by CREB [56, 77, 78] and abolition of BDNF up-regulation has been observed in chronic desipramine treated CREB deficient mice [57]. It plays a key role in therapeutic efficacy [44] of clinically successful serotonergic antidepressants [65, 196, 200, 208, 225, 242] particularly, the SSRIs [230, 331]. CREB plays a key role in antidepressant activity of other serotonergic agents acting through 5-HT_4, 6, 7_ receptors, linked to cAMP cascade [77] or through 5-HT_2_ receptor mediated by calcium activated protein kinase [95] (Fig. **2**). It is also found to play a role in antidepressant-like effects of various drugs of other pharmacological classes, such as phosphodiestrase inhibitors [230] and kappa-opioid receptor antagonist [183]. The noradrenergic mediated regulation of CREB gene is also reported [159]. Viral-mediated increases of CREB in nucleus accumbens (NAc) induced depressive states in rodent FST [247, 309]. Using transgenic mice it was proved that inhibition of CREB in nucleus accumbens produces antidepressant-like effects in learned helplessness paradigm [228]. Thus CREB, is well documented as a neurotrophic factor of depression [221] and we presently intend to view it as downstream target among the various others in the serotonergic system. 5-HT_6_ (and even 5-HT_7_) receptors (discussed above) are G-protein linked and positively coupled to the adenylate cyclase–cAMP system. Activation of these receptors eventually lead to the triggering of CREB [215, 263, 262]. It is noteworthy that 5-HT induces phosphorylation of CREB in HEK 293 cells which have 5-HT_6_ and 5-HT_7_ and expressing the 5-HT transporter (SERT) [144]. Fluoxetine in combination with olanzapine supresses pCREB in rats [131]. Fluoxetine in addition to 5-HT release, exhibited a neuroprotectant effect in neural stem cell culture involving activation of CREB site of c-FLIP promoter region spanning nucleotide which may be mediated by phosphatidylinositol-3-kinase-dependent pathway [51]. Stress is known to activate CREB in the nucleus accumbens and several other regions thereby mediating depression like behaviour [44]. Chronic Citalopram treatment inhibited the CREB phosporylation in female rats [158] and prevented the chronic stress induced CREB mRNA expression in dorsal raphae nucleus of rat [1] and a similar effect was observed with fluoxetine [317]. Glutamate release has been shown to influence the 5-HT_6 _[70] and 5-HT_7_ [32] receptor mediated activation of CREB/BDNF. pCREB is an immediate-early gene transcription factor associated with changes in synaptic efficacy and neuroanatomy in the hippocampus, prefrontal and piriform cortex. Agents which ultimately activate CREB have therapeutic potential in depression. In short the current research has revealed intriguing aspects which imply that strategies that exploit region specific differences in upstream factors, or those which target specific CREB-regulated genes, can contribute to the treatment.

### Serotonin Transporters: 5-HTT/ SERT

In the last two decades, molecular biochemistry [165, 166, 170] and extensive gene [205] based studies on different human population [110, 150, 275, 333] using advanced techniques including brain imaging [43, 114, 119, 136, 253] have been initiated in an attempt to manage human depression. Located on the axolemma outside the synaptic junctions [338], 5-HT transporters are the major sites of action of SSRIs. Studies using animal and *in vitro* simulations pertaining to serotonergic depression are presented as follows. 5-HTT was richly evident in brain stem raphae nuclei of mice [18], hippocampus in primates and rats and in specific subregions of amygdala [232, 236]. Northern blot analysis indicated that 5-HTT gene expression is suppressed in the 5-HT depleted state [168] indicating a trophic role for 5-HT. BDNF modulates 5-HTT in B-lymphocyte cell line and represents a reliable *in vitro* model to examine the functional regulation of 5-HTT by neurotrophins [217]. Fluoxetine treatment down-regulates the expression of many proteins involved in the multiple kinase pathways determining 5-HTT regulation [254] and is shown to decrease the 5-HTT expression in rat dorsal raphae nucleus [233]. 

Developmental loss of 5-HTT produces altered behaviour in models of depression associated with reduced 5-HT neurons and decreased firing rate in the dorsal raphae nucleus [169]. In addition 5-HTT deficient mice have greater levels of extra-cellular 5-HT [198]. Repetitive electro-convulsive shock increases 5-HTT protein expression in the rat frontal cortex probably as a compensatory mechanism against the enhanced ECS induced 5-HT release in presynaptic terminals [274]. In TST, fluoxetine was ineffective as an antidepressant in 5-HTT knockout mice [130] and the disruption at the ‘C’ terminus (in 5-HTT mutant mice) leads to increased duration of immobility [337]. Maternal separation induced increased immobility in rat FST was mediated by decreased hippocampal 5-HT and raphe expression of 5-HTT mRNA [162]. The influence of genetic variation in 5-HTT and its influence on emotional traits have been recognized throwing new insights in understanding the genetic basis of depression [18, 113,114, 265]. To summarize, the blockade of re-uptake mechanism by inhibiting the transporter proteins increases the synaptic concentration of 5-HT. This has been a successful strategy providing us with prototypical antidepressants. 

## OTHER 5-HT RELATED MECHANISMS OF RECENT FOCUS

### Neurokinin Receptors and Substance P

Neurokinin receptor (NK-1) and its endogenous ligand substance P had been implicated in depression. Occurrence of this receptor system in the limbic regions of the brain was the initial spark which kindled behavioural assays with NK-1 receptor antagonists in normal [264] and in transgenic animals [28, 266]. Substance P [129] itself is a stress mediator and its inhibitory effects on serotonergic neurons are mediated by GABA neurons [178] and chronic antidepressant treatment reduces its levels in depression related brain areas [279]. Antagonists at central Substance P receptors (MK-869) are reported to have therapeutic role in depression, with novel mechanism of action [155]. Acute and chronic treatments of CP-96345 an NK-1 receptor antagonist increased neuronal firing in serotonergic neurons of the raphae nucleus and chronic treatment caused tonic activation of postsynaptic 5-HT_1A_ receptors [111]. NK-1 receptor antagonists increased neuronal firing in the norepinephrine neurons in locus ceruleus [202] were these receptors are most expressed [49] and interestingly, such an effect also influenced neuronal firing in the 5-HT neurons [97, 267]. A comparative study in NK-1 receptor deficient mice and fluoxetine/ L-000760735 (a substance P antagonist) treatment indicated that neurofilament alteration and synaptic remodeling contribute to the antidepressant actions of the tested drugs [104]. An exhaustive enzyme, protein and molecular neurochemistry based study on NK-1 receptor knockout mice indicated many changes, analogous to antidepressant treatment [220]. The NK-1 receptor antagonist GR205171, (though inactive by itself) potentiated the antidepressant-like effects of SSRIs in mice FST [50]. Many reviews on NK-1/Substance P [266], classified the NK-1 receptor antagonists as antidepressants and emphasized the requirement of specific ligands and battery of behavioural assays to establish such a novel class of antidepressants [89, 123, 124, 205, 290]. Their association with serotonergic system [2, 290] was also reported. In contrast to the preclinical studies, which have demonstrated antidepressant prospects (with an alleged involvement of the serotonergic system), the NK-1 receptor antagonists have failed in the clinical studies [2,7,261] strongly opposing the notion to consider them as antidepressant target.

### Corticotrophin Releasing Factor 

These constitute a family of peptides and are involved in stress response. The antidepressant-like effects of CP-154,526 (CRF-1 antagonist) were demonstrated in animal models viz. reversal of escape deficits in rat learned helplessness [189] and off late in olfactory bulbectomised rats [137]. Direct administration of CRF into rat dorsal raphae nucleus alters 5-HT release [250]. Antidepressant-like activity demonstrated by few CRF-1 receptor antagonists like R 121919 and DMP 696 [231]. SSR125543, a selective CRF-1 receptor antagonist increased swimming behaviour in Flinders Sensitive Line rat (genetic animal model of depression) [235]. The rat FST, itself can model the influence of CRF on 5-HT neurotransmission [64]. These studies advocated this entire pharmacological class as antidepressants. Direct administration of CRF into rat dorsal raphae nucleus alters exploratory behaviour and serotonergic gene expression [53]. Hence there is a temptation to consider antagonism of CRF-1 receptor to be beneficial in depression and the existence of functional interaction with serotonergic system.

### Cocaine- and Amphetamine-Regulated Transcript 

Cocaine-and amphetamine-regulated transcript (CART), a vesicular neuropeptide [66] expressed in the limbic system [73,74,154,133] is a component of hypothalamic-pituitary axis [93, 287] and is associated in the pathogenesis and treatment of depression. It can presumably act as co-transmitter/neurotransmitter to augment the antidepressant-like effects [237] and reversibly influence many other nuclear mechanisms including CRF/AVP [286], BDNF/CREB [325], extra cellular signal regulated kinase [160] and neurotransmitters including 5-HT [277, 287, 306]. CART elevates extracellular 5-HT in both the rat dorsal raphae nucleus and nucleus accumbens supported by the existence of CART receptors responsible for the depolarization-dependent release [179]. This implies a serotonergic mechanism behind the antidepressant action of CART. However, the influence on locomotion might mask the antidepressant effects while screening in animal models. 

### Arginine Vasopressin 

Arginine vasopressin 1B (AVP-1B) receptor antagonist due to its effects on glucocorticoid and mineralocorticoid receptors gave the first indication involving this system in pathophysiology of depression [85]. SR149415, the selective (and orally active) AVP-1B receptor antagonist exhibited antidepressant-like effects in FST [102] and olfactory bulbectomy [137] models in rats. Fluoxetine decreased AVP release from rat hypothalamic organ culture [6] and attenuated territorial aggression in male coral reef fish [244] and it was concluded that behavioural effects of SSRI (fluoxetine) are partly mediated through Arginine vasotocin system/ vasopressin [273]. Maternal separation, induced depressive behaviour in male rats which is mediated by changes in hypothalamic 5-HT and arginine vasopressin [315]. Further research is essential to confirm its role in serotonergic depression.

### Glutamate Receptor

The recently reported antidepressant effects of N-methy-D-aspartate (NMDA) antagonist ketamine [23, 182, 335] had emphasised the importance of glutaminergic system (including the metabotropic glutamate receptor, mGlu-R) as a candidate mechanism in depression. In the recent past, the involvement of glutamate (excitatory neurotransmitter) pathway [157, 163, 246, 283] and the cross talk of glutaminergic (NMDA) and serotonergic (5-HT_1A/2A_) systems (mainly in the prefrontal cortex) has been implicated in molecular basis of depression [334]. It was found that chronic antidepressant treatment modulates 5-HT turnover in prefrontal cortex in response to phencyclidine, a NMDA antagonist [71]. The antidepressant (and anticonvulsant) activity of NMDA receptor antagonists like MK-801 was associated with increase in hippocampal 5-HT levels [288]. Modulation of metabotropic glutamate receptors have been reported to influence neuronal plasticity and release of neurotransmitters including 5-HT [288, 324]. Recognising the importance of neurogenesis in depression, a cell (progenitor) proliferation study indicated that LY379268 (mGlu2/3 receptor agonist) synergised effects of fluoxetine, implying the association of glutamate and serotonergic pathway in depression [199]. Based on the results from rat FST, the combination of conventional antidepressants (including SSRI and SNRI) and NMDA receptor antagonists was reported to be beneficial in depression [259]. It has been spectulated that differential regulation of distinct glutaminergic afferents on the dorsal raphae nucleus neurons (specific) underlies the behavioural trait of rat in the FST, the widely used (and discussed) model of depression [151]. The alpha-amino-3-hydroxy-5-methyl-4-isoxazolepropionic acid (AMPA) knockout mice was shown to exhibit disturbed glutamate homeostasis and dercreased 5-HT levels, representing a model of depression and as well, the intercommunication of glutaminergic-serotonergic systems [52]. Hence, it was observed ligands of glutamate receptor (NMDA, AMPA and mGlu-Rs) exhibited antidepressant-like effects which are partly dependent on the serotonergic system. However this concept has been of very recent fous and much of the results are still awaited.

## NEURONAL PLASTICITY

Several reports in recent past have correlated depression with alteration in neuronal plasticity and recognised the role of serotonergic system [87, 171] and its receptor subtypes [67]. Neuronal structural plasticity is observed in animal models of depression and antidepressants are known to prevent it [46]. When co-administered with olanzapine, (fluoxetine) modulates neuronal plasticity involving the fibroblast growth factor-2 [190]. Fluoxetine when treated alone restored neuronal plasticity related hippocampal alterations in diabetic mice [17] and increases the expression of polysialylated form of the neural cell adhesion molecule (PSA-NCAM) and synaptophysin (through 5-HT_3_ receptors), which are involved in synaptic remodeling and density, respectively [311]. Thus we can observe that efficacy of serotonergic antidepressants is attributed to modulation of neuronal plasticity.

## ANTIDEPRESSANT ASSAYS

As we have observed, several techniques like immunohistochemistry and radioisotopic studies have identified the receptors in the dynamic neural correlates of depression and it is the prerogative initial step in any drug discovery program. Subsequently, interaction studies with established antidepressants, specific ligands which modulate the activity of target directly or indirectly and usage of knockout animals have been the best methods to mechanistically study the *in vivo* effects of the substance under test. While subjecting a selective ligand to an interaction study (against depression as in this case), two pieces of information can be obtained, (1.) apparent involvement of a particular target (say a receptor or enzyme) in the disorder represented by the model in which it is screened and (2.) usefulness of the particular model in further screening molecules of the same (new) pharmacological class or in short predictive validity of the model, for newly identified target. The present review is intended to serve as the design of pharmacological/genetic studies for screening newer serotonergic antidepressants before taking them to testing on humans. The test species varied from mice to primates. Of all behavioural tests FST, TST (tests of immobility), foot shock (learned helplessness test), and reversal of olfactory bulbectomy induced behavioural deficits have been bested suited to assess the antidepressant-like effects (see Table **1**). There is a obvious lack of selective/ specific ligands, as mentioned in most of the earlier review and research reports and it remains a limiting factor for conducting interaction studies in animal models. On the other hand, *in vitro* studies especially those utilizing neural stem cell culture, and B-Lymphocyte cell lines have significantly helped us to understand the cellular mode of action, but results from such studies have always been correlated with behavioural studies either conducted by same research group or elsewhere. mRNA expression has been the central concept behind the entire research carried to unravel the antidepressant mechanisms. While arriving at the identification of the neuronal target, mRNA expression has been the first step linking the target and manipulative procedure.

Though the exact representation is questionable, it is the knockout animals which had provided the cardinal data and significance to the antidepressant screening process. The simplified premise for knockout studies is that neurobehavioural/ biochemical/ anatomical alteration presumably noticeable (in drug/situation induced conditions) in the absence of particular receptor is expected to furnish information on its (receptor) functionality as a component of the molecular disease state. With the advancements in biotechnology, practically every target (as observed above) can be genetically ablated in test systems (mainly mice). Observation of the entire neural picture including receptor systems, secondary messengers, transporter activation, neurotransmitter release, modulatory proteins, transcriptional factors, gene expression in different areas of the brain in the animals under a state of depression (drug induced/situational/ genetically manipulated) are required for complete understanding of antidepressant drug action. This can be essentially supported by the behavioural data. This list is not complete as other factors such as species variability and extrapolation of results to humans is an even more complex task. Hence it is observed that most of the approaches in modeling and managing depression are somehow influenced by the serotonergic system. However, there is still a need for animal models of antidepressant drug action that selectively screen and support the involvement of the other peptides namely the CART, calcineurin and AVP.

## AN IDEAL BEHAVIOURAL TEMPLATE: SSRI

From the above progress report we can clearly observe the 5-HT neurotransmitter system can be considered as the vital system controlling the internal homeostatic mechanism behind depression. This review drew our attention towards the ‘*blockbuster’* antidepressant fluoxetine and its pharmacological class SSRI, which were extensively studied in the past 2 decades. Every proposed/established serotonergic neuronal target is influenced by fluoxetine (Table **2**) and almost all of the interaction (Pharmacological) and/or knockout (genetic) studies have been conducted with fluoxetine (or other SSRIs), as reference standard or an agent for potentiating antidepressant-like effects of test compounds. The behavioural effects have been attributed to various mechanisms including upregulation of tryptophan hydroxylase [15] and thus there is a temptation to devote the success of protypical serotonergic antidepressants like fluoxetine towards their action on multiple 5-HT receptors, their downstream target proteins (enzymes and receptors) and even on gene expression with an exceptional balance. The pressures on the antidepressant drug discovery (based on the current scenario) will include but not limited to the requirements such as early onset, treatment resistance, control over the entire symptom cloud, effectiveness in comorbid cases and most importantly, least adverse effects. Understanding the behavioural manifests of depression as a consequence of, neurogenesis, neuronal proliferation, plasticity and neurotransmitter release [223] would help us to overcome most of the above mentioned pressures, if not all. Antidepressant effects when mediated separately through each of the above target had a characteristic benefit and interaction studies designed on specific animal models have indicated the possible outcomes of such treatments in humans. In conclusion, a multi-target approach relying mainly on serotonergic system can be the dictum for developing novel antidepressant drugs. With an extensive usage of interaction and knockout animals based assays, a template for behavioural analysis (consequently biochemical analysis) can be designed based on SSRI (especially fluoxetine) drug discovery. Such an approach will definitely help us providing better ‘serotonergic’ antidepressant molecules (safety profile dependent) for trials in the clinic.

## Figures and Tables

**Table 1:** Serotonin Receptors, their Downstream Targets and Screening Methods: Role in Depression

Target	Type of Modulation for Antidepressant Outcome	Method of Assessment (*In Vivo/ In Vitro* Animal Models)
5HT_1A_	Selective Agonism (Full/Partial )	FST, TST, 8-OH-DPAT induced hyperthermia, OBX, learned helplessness, chronic stress, electrophysiological analysis of hippocampal slices and knockout studies.
5HT_1B_	Antagonism	Schedule induced polydipsia, FST, TST, knockout studies and 5-HT release assessment
5HT_2A_	Antagonism	FST,TST, OBX, mRNA expression and differential-reinforcement-of-low-rate 72-sec behaviour
5HT_2C_	Agonism	FST, OBX, operant schedule, electroencephalography and stress induced anhedonia
5HT_3_	Antagonism	FST, learned helplessness, knockout studies and electrophysiological studies.
5HT_6_	Agonism	FST, TST and protein expression
5HT_7_	Antagonism	FST, TST, REM pattern analysis and knockout mice
BDNF/Trk-B receptor	Upregulation	FST, TST, foot shock induced learned helplessness, stress induced depression, OBX, stem cell culture, mRNA levels and knockout studies
CREB	Downregulation	FST, learned helplessness, chronic stress models, stem cell culture and knockout studies
5-HTT/SERT	Inhibiton	FST, knockout studies, maternal separation induced depression, expression in B-Lymphocyte cell line and electroconvulsive shock model
CRF	Antagonism	FST, learned helplessness, OBX and flinders sensitive line rat.
Substance P/NK-1 receptor	Antagonism	FST, neural architechture studies, protein expression and knockout mice
AVP	Antagonism	FST, OBX, maternal separation and territorial aggression in coral reef fish
cFOS	Supression	Stress induced models and expression studies
CART	Activation	5-HT level assessments and protein expression studies
Calcineurin	Activation	TST and expression studies

The table hints the overall status of various probable targets related to the serotonergic system and the screening methods which have been used to correlate them with depression.

**Table 2:** Multiple Neuropharmacological Mechanisms of Fluoxetine Making it an Ideal Antidepressant

Target	Screening Modes	Mechanism*	References
5HT_1A_	Behabioural assays and *in vitro* binding studies in mouse brain and patch clamp.	Postsynaptic receptor activation. Alters responsiveness of receptor-mediated GIRK currents.	[59,125]
5HT_1B_	Behavioural assays and mRNA expression studies.	Down regulation, partial agonist accelerated onset of antidepressant effects of fluoxetine.	[10, 128, 226]
5HT_2A_	Arachidonic acid upregulation study.	Antagonistic action.	[251]
5HT_2C_	Behavioural assays, arachidonic acid upregulation and transcription studies.	Competitive and reversible antagonism. Alters pattern of 5-HT_2C_ transcript editing and potentiates the effect of agonist.	[48,55, 229,251]
5HT_3_	Behavioural assays, patch clamp and expression studies.	Inhibits the peak 5-HT current, potentiates antagonism and modulates PSA-NCAM and synaptophysin in mPFC.	[82, 252, 255, 311]
5HT_6_	Behavioural assays.	Agonistic action.	[291]
5HT_7_	Receptor binding study.	Down regulates receptor binding site	[285]
BDNF	Behavioural assays,mRNA expression studies and knockout models.	Upregulation. (duration of treatment dependent)	[24, 58, 69, 115]
CREB	Expression analysis and neural stem cell culture studies.	Upregulation.	[51, 317]
5-HTT	Knockout models, mRNA and gene expression studies.	Inhibitor and reduces gene expression.	[130, 233, 254]
AVP	Behavioural assays and neurotransmitter release studies.	Decreased release and down regulation.	[6, 137,244, 273]
Multiple heat shock protein, neurofilaments and related proteins.	Assessment of microanatomy and gene expression.	Synaptic remodeling favouring antidepressant action.	[131]
FGF-2	Ribonuclease protection assay and western blot analysis	Upregulation (when co-administered with olanzapine)	[190]
14-3-3zeta mRNA, Tyrptophan hydroxylase	PCR and western blot analysis on RBL-2H3 cells	Upregulation.	[15]

* The mechanisms are discussed in detail in the text.
